# Effect of Probiotics and Therapeutic Additives on the Osmolality of Pasteurized Donor Human Milk

**DOI:** 10.7759/cureus.106379

**Published:** 2026-04-03

**Authors:** Laxman Basany, Abid Ali, Naga Priyanka G Gandrakota, Ajay B Kulkarni, Vinay Batthula, Mahevish Tabassum, Roja Aepala, Harini Manjunath

**Affiliations:** 1 Neonatology, Ankura Hospital for Women and Children, Hyderabad, IND; 2 Neonatology, Ankura Hospital for Women and Children, Delhi, IND; 3 Neonatology, Ankura Hospital for Women and Children, Visakhapatnam, IND; 4 Dietetics, Ankura Hospital for Women and Children, Hyderabad, IND; 5 Pediatrics, Rainbow Children’s Hospital, Hyderabad, IND; 6 Pharmacology, Independent Research, Bengaluru, IND

**Keywords:** additives, human milk, hyperosmolar, necrotizing enterocolitis (nec), neonate, osmolality, pasteurized donor human milk, preterm, probiotics, water soluble oral contrast

## Abstract

Aim: This study aims to examine changes in the osmolality of pasteurized donor human milk (PDHM) following the addition of probiotics and commonly used neonatal additives.

Methods: The osmolality of PDHM was measured at 10 minutes using a calibrated osmometer by a technician blinded to the probiotics and additives used in the study. The osmolality of 5 mL of PDHM was measured after the addition of four different probiotics and commonly used additives, including caffeine, cephalexin, domperidone, esomeprazole, fluconazole, furosemide, ibuprofen, lansoprazole, levetiracetam, paracetamol, phenobarbitone, sildenafil, and ursodeoxycholic acid, administered separately in therapeutic doses. The osmolality of the oral iodinated non-ionic contrast agent (Omnipaque) was measured after 1:2 and 1:4 dilution with sterile water. The volume of PDHM required to be added to each additive to keep the osmolality below 450 mOsm/kg, as recommended by the European Society for Pediatric Gastroenterology, Hepatology and Nutrition (ESPGHAN), was calculated.

Results: The mean osmolality of PDHM was 256 mOsm/kg, which, on reconstitution with most additives, exceeded 450 mOsm/kg. The osmolality of pure additives ranged from 161 to >2000 mOsm/kg. There was a significant increase in the osmolality of PDHM beyond the recommended 450 mOsm/kg with most additives, except caffeine, cephalexin, esomeprazole, fluconazole, furosemide, lansoprazole, and sildenafil. A maximum increase in osmolality to 1723 mOsm/kg (range 260-1723 mOsm/kg) was observed with probiotics. Domperidone, ibuprofen, levetiracetam, paracetamol, phenobarbitone, and ursodeoxycholic acid increased the osmolality beyond 450 mOsm/kg. The osmolality of the iodinated non-ionic contrast agent (Omnipaque), when diluted with sterile water in a 1:4 ratio, was 461 mOsm/kg.

Conclusion: The addition of probiotics to PDHM increases osmolality and requires appropriate dilution to maintain levels below the recommended 450 mOsm/kg. Therapeutic additives such as domperidone, ibuprofen, levetiracetam, paracetamol, phenobarbitone, and ursodeoxycholic acid also increase the osmolality of PDHM and require appropriate dilution. Future research should focus on developing probiotics and additives with lower osmolality to improve safety in neonates.

## Introduction

Breast milk is the appropriate nutrition for all neonates because of its nutritional, bioactive, and immunological factors, which provide both short-term and long-term benefits. Pasteurized donor human milk (PDHM) is recommended as the optimal alternative in the absence of the mother’s own milk [1,2]. However, preterm neonates have higher nutritional requirements and need to be supplemented with macronutrients, minerals, and vitamins that are added to expressed breast milk (EBM) in the form of fortifiers and supplements [3]. Apart from these nutritional supplements, probiotics, caffeine, cephalexin, domperidone, esomeprazole, fluconazole, furosemide, ibuprofen, lansoprazole, levetiracetam, paracetamol, phenobarbitone, sildenafil, and ursodeoxycholic acid are often used for therapeutic management in neonates. However, these additives can increase the osmolality of EBM and can cause feeding intolerance, delayed gastric emptying, and necrotizing enterocolitis (NEC) [4,5]. Hence, European Society for Paediatric Gastroenterology, Hepatology and Nutrition (ESPGHAN) and the American Academy of Pediatrics (AAP) recommend that the osmolality of enteral feeds for neonates should not exceed 450 mOsm/kg [6,7].

Probiotics are increasingly used in preterm neonates to reduce the risk of dysbiosis [8]. They decrease the time to reach full feeds, late-onset sepsis (LOS), all-cause mortality, and NEC in preterm and very low birth weight (VLBW) neonates [9-11]. ESPGHAN recommends the use of probiotics for very preterm neonates (<32 weeks of gestation) [6]. However, probiotics can increase osmolality and lead to adverse effects [12].

Medications such as oral caffeine are commonly used in preterm infants to treat apnea of prematurity and to reduce the risk of bronchopulmonary dysplasia (BPD). Cephalexin is commonly used as chemoprophylaxis in neonates with hydronephrosis to prevent urinary tract infections. Domperidone is a prokinetic agent frequently used to treat gastroesophageal reflux, despite limited evidence supporting its efficacy. Proton pump inhibitors (PPIs) such as esomeprazole and lansoprazole are often used in the treatment of gastroesophageal reflux disease (GERD) [13]. Ibuprofen and paracetamol are widely used for the medical closure of patent ductus arteriosus (PDA) in neonates. Anticonvulsants such as levetiracetam and phenobarbitone are administered orally for maintenance therapy. Oral sildenafil for pulmonary hypertension is generally preferred over the parenteral route, as it is associated with a lower incidence of significant adverse effects, including systemic hypotension. Ursodeoxycholic acid is used in the management of neonatal cholestasis and inspissated bile syndrome to promote bile flow and improve cholestasis [14,15]. Omnipaque is a non-ionic iodinated contrast agent commonly used in gastrointestinal contrast studies [16]. Several studies have shown that adding medications to human milk increases feed osmolality and recommend appropriate dilution to prevent hyperosmolality.

However, evidence on the osmolar effects of probiotics and medications, including oral antibiotics, domperidone, esomeprazole, fluconazole, furosemide, ibuprofen, lansoprazole, levetiracetam, paracetamol, phenobarbitone, sildenafil, and ursodeoxycholic acid, when added to PDHM, is limited.

The primary objective of our study was to evaluate the osmolality of various additives mixed with human milk; Omnipaque was included as a specific exception. Oral contrast studies in neonates with suspected intestinal obstruction are performed while the infant is kept nil per os, and the contrast agent is routinely diluted with sterile water prior to administration.

Given its frequent use in neonatal practice, we assessed the osmolality of this oral contrast agent, alongside other commonly used neonatal additives, to identify a dilution that would maintain osmolality within acceptable limits and thereby improve safety in this vulnerable population. Accordingly, serial dilutions of Omnipaque with sterile water were prepared to determine the dilution at which a safe osmolality could be achieved.

Therapeutic doses of probiotics and medications were added to PDHM in serial dilutions to determine the appropriate dilution required to keep the osmolality below 450 mOsm/kg.

## Materials and methods

This study was approved by the Ankura Hospital Ethics Committee. Osmolality was measured using a calibrated osmometer (OSMO 1, Advanced Instruments, Norwood, Massachusetts) based on the freezing point depression method, with a precision of 2 mOsm/kg. A standard osmolality solution provided by the manufacturer was used for calibration. All measurements were conducted at the NeoKare Nutrition Laboratory in Redditch, United Kingdom. Transporting PDHM from our center was not feasible due to challenges in maintaining the cold chain, storage requirements, regulatory compliance for biological fluids, and other logistical constraints. Therefore, PDHM sourced from the NeoKare Nutrition Milk Bank in the United Kingdom was used, while the therapeutic additives and probiotic preparations evaluated were those commonly used in neonatal intensive care units in India. All tests were performed by a technician blinded to the probiotics and additives used in the study. PDHM stored at −20 °C was thawed in tepid water immediately prior to testing. The composition of PDHM was as follows: approximately 71.96 kcal per 100 mL of energy, with a protein content of 0.97 g/dL. The total carbohydrate content was 7.71 g/dL, comprising 7.23 g/dL of lactose and 0.48 g/dL of oligosaccharides. The fat content was 4.14 g/dL.

The osmolality of PDHM was measured at 10 minutes after reconstitution with probiotics and additives, namely PB1 Darolac sachet (Aristo Pharma); PB2 Pro GG drops (Aristo Pharma); PB3 Pro GG sachet (Aristo Pharma); PB4 Rescunate sachet (Tablets India Ltd); caffeine (Cafirate syrup, 20 mg/mL, Cipla); cephalexin (Sporidex drops, 100 mg/mL, Sun Pharmaceuticals); domperidone (Domstal suspension, 1 mg/mL, Torrent Pharmaceuticals); esomeprazole (Esomac sachet, 10 mg per 1 g sachet, Cipla); fluconazole tablet (Forcan 50 mg, Cipla); furosemide (Furoped syrup, 10 mg/mL, Samarth Pharma); ibuprofen syrup (Ibugesic syrup, 20 mg/mL, Abbott Healthcare); lansoprazole (Lanzol Junior 15 mg DT, Cipla); levetiracetam (Levepsy syrup, 100 mg/mL, Cipla); paracetamol (P 100 drops, 100 mg/mL, Apex Pharmaceuticals); phenobarbitone (Gardenal syrup, 20 mg/mL, Abbott Healthcare); sildenafil tablet (Suhagra 25 mg, Cipla); and ursodeoxycholic acid (Udcament syrup, 25 mg/mL, Akums Drugs & Pharma). The iodinated non-ionic contrast agent Omnipaque (GE Healthcare) contains iohexol, with an iodine concentration of 350 mg/mL.

A 2 kg preterm neonate was used as the reference to calculate doses of all additives, which were added to 5 mL aliquots of PDHM. When the osmolality of a pure additive exceeded 2000 mOsm/kg, which is the maximum measurable range of the osmometer, it was serially diluted with PDHM, and measurements were repeated until the osmolality was below 450 mOsm/kg. All measurements were performed in duplicate and averaged. If the difference between readings exceeded 2 mOsm/kg, a third measurement was taken, and the two closest values were averaged. The volume of PDHM required to maintain osmolality below 450 mOsm/kg was calculated for each additive.

## Results

The mean osmolality of PDHM measured was 256 mOsm/kg (range 255-257 mOsm/kg). The composition of the probiotics used in the study is presented in Table 1. 

**Table 1:** Composition of probiotics CFU: colony-forming unit; PB: probiotic; PB1: Darolac sachet; PB2: Pro GG drops; PB3: Pro GG sachet; PB4: Rescunate sachet.

Variables	PB1	PB2	PB3	PB4
Strain	Lactobacillus acidophilus, Lactobacillus rhamnosus, Bifidobacterium longum, Saccharomyces boulardii	Lactobacillus rhamnosus GG	Lactobacillus rhamnosus GG	Bifidobacterium breve M-16V
Strength	1.25 billion CFU/g	1 billion CFU/0.2 mL	5 billion CFU/0.5 g	0.5 billion CFU/0.5 g

**Figure 1:**
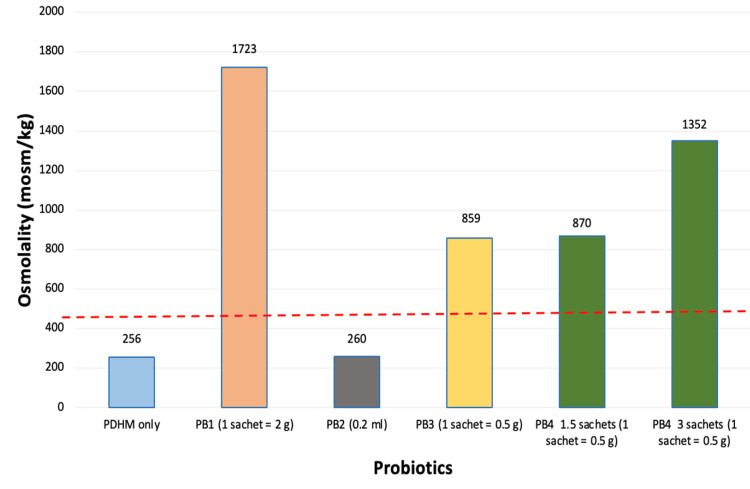
Osmolality of pasteurized donor human milk (PDHM) with probiotics The red dotted horizontal line indicates the safety threshold of 450 mOsm/kg; values above this line exceed the recommended safety limit. CFU: colony-forming unit; PB: probiotic; PDHM: pasteurized donor human milk; PB1: Darolac sachet; PB2: Pro GG drops; PB3: Pro GG sachet; PB4: Rescunate sachet.

**Figure 2:**
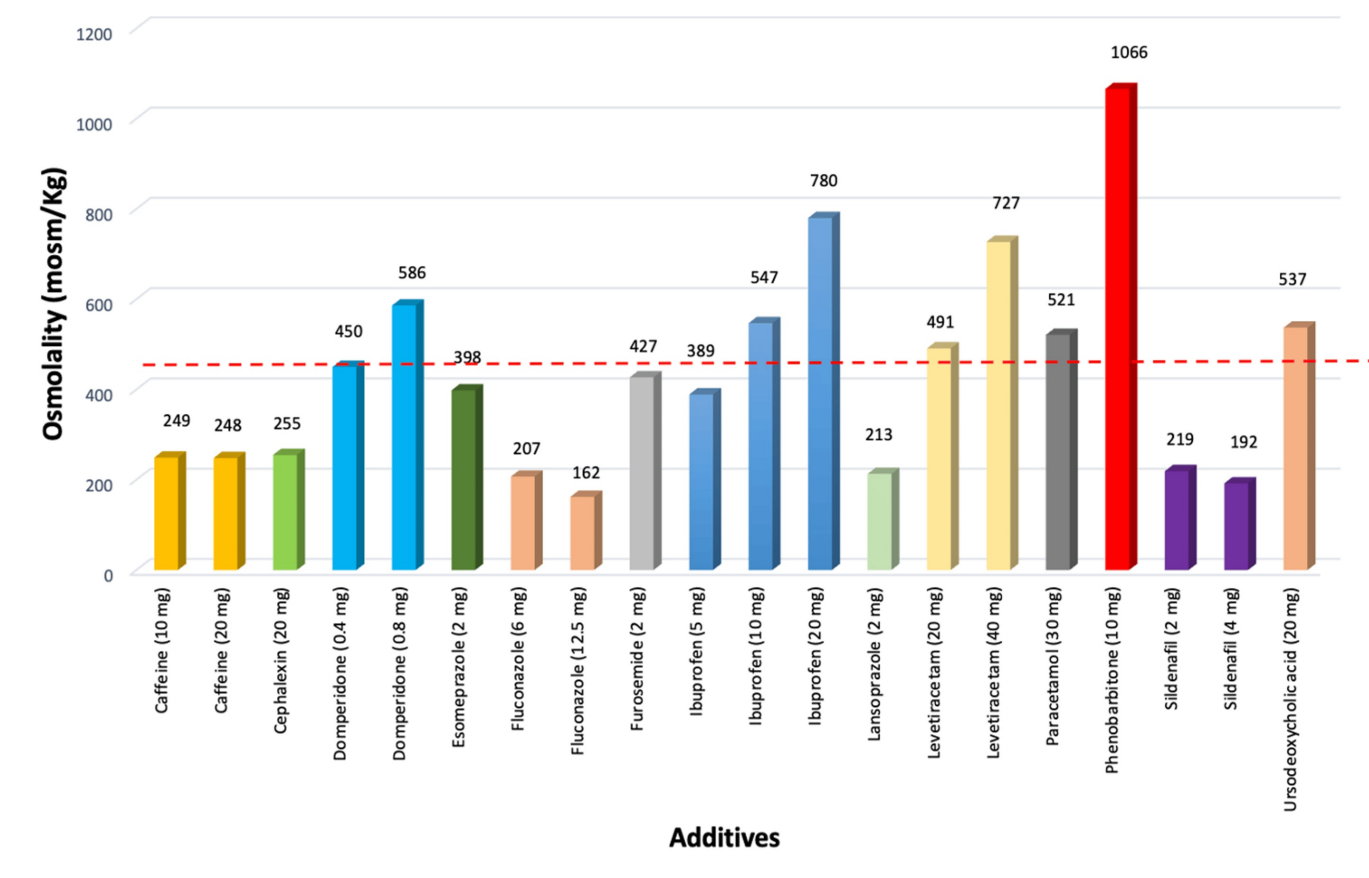
Osmolality of PDHM with additives The red dotted horizontal line indicates the safety threshold of 450 mOsm/kg; values above this line exceed the recommended safety limit. PDHM: pasteurized donor human milk.

The osmolality of PDHM following the addition of probiotics and medications, measured at 10 minutes, is shown in Figure 1 and Figure 2, respectively. 

The osmolality of PDHM increased beyond 450 mOsm/kg with all additives except caffeine, cephalexin, esomeprazole, fluconazole, furosemide, lansoprazole, Pro GG drops (PB2), and sildenafil. The addition of domperidone, ibuprofen, levetiracetam, paracetamol, phenobarbitone, and ursodeoxycholic acid to PDHM increased the osmolality to levels exceeding 450 mOsm/kg.

The highest increase in osmolality of PDHM was observed with probiotics, where the osmolality increased to 1723 mOsm/kg (range 260-1723 mOsm/kg). The osmolality of PDHM and sterile water after the addition of probiotics is shown in Table 2. 

**Table 2:** Osmolality of PDHM and sterile water with probiotics Probiotics, being hyperosmolar, are often mixed with sterile water when the feed volume is <5 mL. Hence, the osmolality of probiotics was measured after dilution with sterile water to determine the minimum dilution volume required to ensure safety. PB1: 1.25 billion CFU/g; PB4: 0.5 billion CFU/0.5 g. PDHM: pasteurized donor human milk, PB1: Darolac sachet (each sachet = 2 g); PB4: Rescunate sachet (each sachet = 0.5 g).

Additive	Diluent	Dose of additive	Mean osmolality in mOsm/kg
PB1	5 mL of sterile water	½ sachet (1 g)	803
PB1	10 mL of sterile water	½ sachet (1 g)	410
PB1	5 mL of PDHM	½ sachet (1 g)	1151
PB4	3 mL of sterile water	1.0 sachet (0.5 g)	412
PB4	5 mL of sterile water	1.5 sachets (0.75 g)	424
PB4	5 mL of sterile water	3 sachets (1.5 g)	935
PB4	10 mL sterile water	3 sachets (1.5 g)	432
PB4	5 mL of PDHM	1.5 sachets (0.75 g)	870
PB4	5 mL of PDHM	3 sachets (1.5 g)	1352

Since Omnipaque is not administered with milk, its osmolality was measured after dilution with sterile water; the osmolality was 553 and 461 mOsm/kg at dilution ratios of 1:2 and 1:4 (contrast:sterile water), respectively, as shown in Table 3.

**Table 3:** Osmolality of Omnipaque with sterile water *Osmolality of Omnipaque is 780 mOsm/kg.

Additive	Quantity	Diluted with 5 mL of sterile water (mean osmolality in mOsm/kg)
Omnipaque*	1.25 mL	461
	2.5 mL	553

The volume of PDHM required to be added to probiotics and other additives to maintain the osmolality below 450 mOsm/kg is shown in Table 4.

**Table 4:** Amount of PDHM required as solvent to keep osmolality below 450 mOsm/kg *Osmolality measured 10 min after reconstitution. PB1: 1.25 billion CFU/g; PB3: 5 billion CFU/0.5 g; PB4: 0.5 billion CFU/0.5 g. PDHM: pasteurized donor human milk; PB1: Darolac sachet; PB3: Pro GG sachet; PB4: Rescunate sachet.

Additive	Strength	Dosage	Amount of PDHM required	Mean osmolality in mOsm/kg*
Caffeine	1 mL = 10 mg	0.5 mL	5 mL	249
		1.0 mL	5 mL	248
Cephalexin	1 mL = 100 mg	0.2 mL	5 mL	255
Domperidone	1 mg per mL	0.4 mL	5 mL	450
		0.6 mL	10 mL	420
Esomeprazole	10 mg per sachet	1 mL (2 mg)	5 mL	398
Fluconazole	1 tablet = 50 mg	1.2 mL (6 mg)	5 mL	207
	(Dissolved in 10 mL sterile water)	2.4 mL (12 mg)	5 mL	214
Furosemide	10 mg per mL	0.2 mL (2 mg)	5 mL	427
Ibuprofen	1 mL = 20 mg	5 mg	5 mL	407
Lansoprazole	15 mg tablet	2 mL (2 mg)	5 mL	213
	(Dissolved in 15 mL sterile water)			
Levetiracetam	1 mL = 100 mg	0.2 mL	10 mL	378
		0.4 mL	15 mL	416
Paracetamol	1 mL = 100 mg	0.3 mL	10 mL	397
PB1	2 g sachet	1/2 sachet	25 mL	434
PB3	0.5 g sachet	1 sachet	20 mL	435
PB4	0.5 g sachet	3 sachets	25 mL	418
Phenobarbitone	1 mL = 20 mg	0.5 mL	30 mL	388
Sildenafil	1 tablet = 25 mg	0.8 mL	5 mL	207
	(Dissolved in 10 mL sterile water)	1.6 mL	5 mL	210
Ursodeoxycholic acid	25 mg per mL	0.8 mL (20 mg)	7.5 mL	418

## Discussion

The study evaluated 4 probiotics and 13 medications that are commonly used in clinical practice in India. In addition to these medications, the osmolality of the contrast agent Omnipaque was measured after dilution with sterile water. There is limited evidence on the impact of probiotics, oral antibiotics, domperidone, esomeprazole, fluconazole, furosemide, ibuprofen, lansoprazole, levetiracetam, paracetamol, phenobarbitone, sildenafil, and ursodeoxycholic acid on the osmolality of PDHM. This study demonstrated that the addition of probiotics and other therapeutic additives to PDHM significantly increases its osmolality, frequently exceeding the safe limits recommended by AAP and ESPGHAN [6,7].

White and Harkavy reported that the addition of medications increased the osmolality of formula milk by up to 300% [17]. Similarly, Ernst JA et al. measured the osmolality of 63 oral and intravenous drug preparations and reported a significant increase in osmolality when these medications were added to formula milk. They reported that feed osmolality was three to four times higher when oral formulations were used compared to the corresponding intravenous preparations [18]. Srinivasan et al. reported increased osmolality with therapeutic additives and recommended appropriate dilution to avoid hyperosmolality [19].

This study demonstrated that the addition of domperidone, ibuprofen, levetiracetam, paracetamol, phenobarbitone, ursodeoxycholic acid, and probiotics (except PB2) increased the osmolality of PDHM to levels exceeding 450 mOsm/kg.

Among the four probiotics tested, PB2 caused the smallest increase in osmolality (260 mOsm/kg), followed by PB3 (859 mOsm/kg), PB4 (1352 mOsm/kg), and PB1 (1723 mOsm/kg), which produced the greatest increase. The carbohydrate (dextrin) content of the probiotic preparations was not disclosed by the manufacturer. PB2 demonstrated the lowest osmolality, likely due to its lower carbohydrate content. Among the probiotics studied, PB1, a multi-strain probiotic, demonstrated the highest osmolality, likely due to its higher carbohydrate content. Amylase present in human milk breaks down probiotic carbohydrates into osmotically active mono- and oligosaccharides, thereby increasing the osmolality. The variation in osmolality among the probiotics may be attributed to differences in the type and composition of carbohydrates present, such as glucose, maltose, maltotriose, glucose tetramers, corn starch, and dextrins [20,21]. The high osmolality of additives may not be attributable to the active drugs themselves, but rather to excipients such as propylene glycol, sorbitol, and other preservatives. The osmolality of PDHM is approximately 20-30 mOsm/kg lower than that of unpasteurized human milk, likely due to the effects of processing and pasteurization [22,23].

Probiotics are routinely administered to infants born at <32 weeks’ gestation. ESPGHAN recommends either Lactobacillus rhamnosus GG or a combination of Bifidobacterium infantis BB-02, Bifidobacterium lactis BB-12, and Streptococcus thermophilus TH-4 [21,24]. The National Neonatology Forum (NNF) recommends the use of multi-strain probiotics in preterm infants <32 weeks or <1500 grams, initiated as early as day one of life and continued until 34-36 weeks postmenstrual age or discharge (whichever occurs earlier), particularly in neonatal units with a high baseline incidence of NEC [25].

Probiotics improve feed tolerance, promote the establishment of beneficial gut microbiota, reduce the time to achieve full enteral feeds, and decrease the incidence of NEC, late-onset sepsis, and all-cause mortality [9-11]. In Japan, probiotics are diluted with 4 mL of distilled water for extremely preterm infants to maintain isosmolarity and ensure safety [26].

The probiotic strain Bifidobacterium breve M-16V (Rescunate, Tablets India) is administered to all preterm infants <32 weeks’ gestation. Due to its high osmolality, the probiotic is diluted with sterile water rather than milk. An initial dose of 1.5 sachets (1.5 billion CFU) of PB4 is administered starting on day two of life. Once enteral feeds reach 50-60 mL/kg/day and are well tolerated, the dose is increased to three sachets (3 billion CFU) per day. Adding 1.5 sachets of PB4 to 5 mL of sterile water resulted in an osmolality of 419 mOsm/kg, which is within the safe range, whereas adding the same amount to 5 mL of PDHM increased the osmolality to 870 mOsm/kg, a level that may be associated with adverse effects. Accordingly, one sachet of PB4 is diluted in 3 mL of sterile water instead of milk to maintain isosmolarity, and this preparation is administered three times daily.

Among the four probiotic preparations commonly used in India, two are routinely prescribed for the management of antibiotic-associated diarrhea (PB1) and infantile colic (PB2). The addition of 1 g (half sachet) of PB1 to 5 mL of sterile water and PDHM increased the osmolality to 803 mOsm/kg and 1151 mOsm/kg, respectively. To maintain osmolality below 450 mOsm/kg, 1 g (half sachet) of PB1 would need to be diluted in at least 10 mL of sterile water or 25 mL of PDHM (Tables 2, 4). The addition of 1.5 and three sachets of PB4 to 5 mL of sterile water increased the osmolality to 419 mOsm/kg and 935 mOsm/kg, respectively. The addition of three sachets of PB4 to 5 mL of PDHM increased the osmolality to 1352 mOsm/kg, indicating marked hyperosmolality. To maintain osmolality below 450 mOsm/kg, three sachets (1.5 g) of PB4 should be diluted in at least 10 mL of sterile water or 25 mL of PDHM (Tables 2, 4).

PB2 (0.2 mL = 1 billion CFU) resulted in the smallest increase in osmolality and can be safely added to 5 mL or smaller volumes of PDHM, whereas PB3 (0.5 g per sachet = 5 billion CFU) should be diluted in at least 20 mL of PDHM to maintain safe osmolality levels (Table 4).

Storage of human milk after the addition of probiotics or therapeutic additives may lead to a further increase in osmolality and a higher risk of microbial contamination. Therefore, standard clinical practice in neonatal units is to prepare such mixtures immediately prior to administration rather than storing them. In accordance with this practice, we measured osmolality immediately after the addition of probiotics and therapeutic additives. Accordingly, probiotics and additives are administered immediately after dilution with milk and are not stored.

Powdered probiotic formulations have higher osmolality than liquid preparations, likely due to excipients present in the powdered form. In addition, multi-strain probiotics available as powders tend to have higher osmolality compared to single-strain probiotic formulations.

The addition of domperidone, ibuprofen, levetiracetam, paracetamol, phenobarbitone, and ursodeoxycholic acid increased the osmolality of PDHM beyond the recommended safe limit and should therefore be diluted in an appropriate volume of milk to maintain osmolality below 450 mOsm/kg. The addition of caffeine, cephalexin, fluconazole, and sildenafil to PDHM did not increase the osmolality beyond 260 mOsm/kg, as shown in Figure 2. Medications available in tablet form did not significantly increase osmolality. Similarly, caffeine did not increase osmolality and can be safely administered enterally.

Esomeprazole, furosemide, and lansoprazole can be safely added to 5 mL of PDHM, as the resulting osmolality remained below the acceptable threshold of 450 mOsm/kg. Paracetamol drops should be diluted with at least 10 mL of PDHM, and ursodeoxycholic acid should be added to at least 7.5 mL of PDHM to ensure that the osmolality remains below 450 mOsm/kg. Although our objective was to assess additives mixed with human milk, Omnipaque was included because of its frequent neonatal use. As Omnipaque is diluted with sterile water prior to administration, its osmolality was measured after dilution and found to be 553 mOsm/kg at a 1:2 dilution and 461 mOsm/kg at a 1:4 dilution (contrast:sterile water). Therefore, a 1:4 dilution is recommended, particularly in preterm infants, to help maintain safer osmolality levels.

An association between hyperosmolar preparations (>400 mOsm/kg) and necrotizing enterocolitis (NEC) has been reported; however, a definitive safe osmolality threshold has not been established [26-29]. Since oral medications commonly used in neonates have high osmolality, greater caution is required, particularly in preterm infants who are at increased risk of NEC. Appropriate dilution of additives to maintain osmolality below 450 mOsm/kg is recommended to enhance safety.

Limitations of the study: Although many probiotic preparations are available in India, we evaluated only a few strains that are commonly used in neonatal practice. PDHM was used in this study and has a lower osmolality compared to unpasteurized EBM. Since unpasteurized EBM has a higher baseline osmolality, a larger volume would be required for dilution to maintain the final osmolality below 450 mOsm/kg compared with PDHM. The PDHM used in this study was obtained from a milk bank in the United Kingdom, and its composition and osmolality may differ from PDHM available in India. This was a laboratory-based investigation designed to allow controlled assessment of changes in osmolality following the addition of commonly used probiotics and therapeutic additives to PDHM; therefore, the findings may not fully reflect in vivo feeding conditions. The potential variability in donor human milk composition, which may influence osmolality measurements, and the limited number of probiotic formulations and therapeutic additives evaluated may affect generalizability. The study did not assess clinical outcomes such as feeding intolerance or necrotizing enterocolitis. Therefore, the results should be interpreted with caution, particularly when EBM is used instead of PDHM.

## Conclusions

Most additives commonly used in neonates are hyperosmolar. Appropriate dilution of these additives to maintain osmolality below 450 mOsm/kg is essential to ensure safety. Parents and caregivers should be educated about the hyperosmolar nature of certain additives and advised on appropriate dilution to minimize the risk of complications. Manufacturers should provide clear information on osmotic strength and recommended dilution to prevent hyperosmolality and reduce the risk of adverse effects on the neonatal gut. Dextrins used as excipients in probiotic formulations are hyperosmolar and should be avoided. Further research should focus on developing probiotics and additives with lower osmolality to improve safety in neonatal use.
